# Substrate system outperforms water-culture systems for hydroponic strawberry production

**DOI:** 10.3389/fpls.2025.1469430

**Published:** 2025-02-12

**Authors:** George Kerrigan Hutchinson, Lan Xuan Nguyen, Zilfina Rubio Ames, Krishna Nemali, Rhuanito Soranz Ferrarezi

**Affiliations:** ^1^ Controlled Environment Agriculture Crop Physiology and Production Laboratory, Department of Horticulture, University of Georgia, Athens, GA, United States; ^2^ Small Fruit Laboratory, Department of Horticulture, University of Georgia, Tifton, GA, United States; ^3^ Controlled Environment Agriculture Laboratory, Department of Horticulture and Landscape Architecture, Purdue University, West Lafayette, IN, United States

**Keywords:** *Fragaria* × *ananassa*, soilless substrate, nutrient film technique, vertical tower, aeroponics, resource use efficiency

## Abstract

Strawberries (*Fragaria × ananassa*) are a globally cultivated fruit crop known for their economic significance and versatility in both fresh markets and processed food industries. Their high consumer demand and market value contribute to substantial profitability for producers. In recent years, due to increasing costs of production and occurrence of extreme weather events, the use of controlled environment agriculture (CEA) and hydroponics for strawberry production has become popular in several Asian, European, and American countries. There are two main types of hydroponic systems: substrate- and water-culture. Substrate-culture systems are the common choice for CEA strawberry production, whereas water-culture systems are usually used for crops like leafy greens and herbs. Both systems have been independently studied for CEA strawberry production, but direct comparisons between them are still limited. The objective of this study was to compare the performance of substrate and water-culture systems for CEA strawberry production regarding yield and resource use efficiencies. ‘Florida Brilliance’ and ‘Florida Beauty’ strawberries were grown in a greenhouse in one substrate-culture, with plants grow in soilless media, and three water-culture systems: nutrient film technique (NFT), vertical tower (stacked nutrient flow), and aeroponics (nutrient misted roots). The system inputs (water, energy, and area) and outputs (yield, biomass, etc.) were quantified during the 129-day experiment. Fruit yield was used to calculate water (WUE), energy (EUE), and area (AUE) use efficiencies. Based on yield and resource use efficiencies, the substrate system performed the best, with the vertical tower system also showing promising performance. The results of this experiment can help growers understand the tradeoffs between hydroponic systems to maximize both profits and sustainability for CEA strawberry production.

## 1 Introduction

Strawberries (*Fragaria × ananassa*) are a widely cultivated and popular fruit crop with active commercial production in approximately 76 countries (Hytönen et al., 2018). China was the largest producer in 2022, with a harvest of 3.35 million metric tons, while the United States was the second largest producer, with a harvest of 1.26 million metric tons (USDA, 2022). In the United States, California (90.6%) and Florida (9.4%) comprise nearly all domestic strawberry production (USDA, 2022). Production from these two states in 2021 was valued at $3.42 billion, a 31% increase compared to the previous year. This increase is due to the rising cost of inputs (fertilizer, equipment, and labor), increased consumer demand, and reduced yields due to abnormal weather (USDA, 2022). Irregular weather is projected to become more frequent and extreme in the coming decades as the effects of climate change continue to develop (Bolster et al., 2023).

Most domestic strawberries are grown using the annual hill plasticulture system. While popular and effective, this system relies heavily on soil fumigants to minimize disease pressure and preserve yields. This reliance on pesticides, increasing input costs, and the effects of climate change have led to rising interest in controlled environment production of strawberries in recent years (Samtani et al., 2019). Controlled environment production, also known as controlled environment agriculture (CEA), aids in insulating crops from the adverse effects of climate change, decreases the need for pesticides, and increases the growing season length for areas without ideal climates. CEA accomplishes these feats by leveraging technology and engineering to optimize the crop’s growing environment. Soilless hydroponic growing systems can further minimize pesticide use while minimizing the use of other inputs like water and fertilizer (Gómez et al., 2019; Wrenn et al., 2023).

CEA strawberry production in the form of low-tunnels, high-tunnels, and climate-controlled greenhouses has been established commercially outside of the United States for many years. South Korea, Japan, the Netherlands, Belgium, France, the United Kingdom, and Italy have produced strawberries commercially in controlled environments for decades (Ahn et al., 2021; Neri et al., 2012; Yoshida, 2013). This controlled environment strawberry industry is also making its way into North America’s greenhouses. A Dutch company recently completed a 29-hectare greenhouse facility for commercial controlled environment strawberry production in Ontario, Canada, the largest such facility on the continent (Hemmes, 2021). Recent advancements in CEA are allowing strawberry farms to be installed indoors in specialized vertical systems where the crops are grown with tightly controlled environmental parameters and sole-source artificial lighting. Since these indoor systems are not reliant on outdoor weather or climate conditions, they can be implemented anywhere. This capability enables food production to be brought closer to consumers, thereby reducing transportation distances and further decoupling our food production infrastructure from the uncertainties of an increasingly unstable environment.

These CEA systems are attractive to many North American growers, with the most popular crops currently being leafy greens, microgreens, and herbs (Van Gerrewey et al., 2021), and most recently growing interest in strawberries. For example, in 2022, a large CEA company announced a partnership with a global leader in the strawberry market to build what will purportedly be the largest indoor vertical farm complex in the world, which will produce millions of kilograms of strawberries annually (Green, 2023; Oller, 2022). The complex will be located in Virginia – a state with minimal commercial strawberry production where 79% of growers operate on 5 acres or fewer (Christman and Samtani, 2019). The state’s strawberry industry could soon see extreme growth thanks to the CEA industry. In addition to the economic impact of this technology, the wider distribution of commercial strawberry production will aid in mitigating the negative effects of extreme weather events and preserve the availability of a valuable, high-demand fruit crop for consumers.

Since greenhouses and vertical farms are more energy-intensive than traditional field production, growers must understand how to minimize their input costs to maximize their business’s profitability. There are many types of hydroponic growing systems that are commonly used in CEA, and these systems can heavily influence growers’ input costs. Hydroponic systems can broadly be divided into two groups: substrate- and water-culture systems. Substrate-culture systems provide a soilless material, usually peat, coco coir, perlite, or some mix thereof, into which the roots can grow. The substrate can then be irrigated and/or fertilized by drip, ebb-and-flow subirrigation, or other methods. In water culture systems, there is no solid substrate, and the roots primarily interact with an aqueous fertilizer solution. This solution can be in constant contact with the roots (statically or dynamically), or the roots can be intermittently exposed to the solution via flowing or spraying/misting.

These two groups of hydroponic systems have been separately studied in several experiments for CEA strawberry production. Within substrate-culture systems, substrate compositions and fertigation solution formulations have been popular avenues of investigation (Cantliffe et al., 2006; Ebrahimi et al., 2012; Linardakis and Manios, 1991; McKean, 2019; Takeda, 1999). In water culture systems, solution temperatures, individual nutrient elemental concentrations, and electrical conductivity (EC) levels have all proven to be common topics (Akon et al., 2018; Caruso et al., 2011; Chow et al., 2004; Economakois and Krulji, 2001; Eissa et al., 2016; Masaru et al., 2016). There have only been a few studies that have generally compared the performance of different hydroponic systems concerning yield (Mohamed et al., 2022; Richardson et al., 2022), and very few have compared the performance of soil or substrate-culture systems with water culture systems (Albaho et al., 2008; Treftz and Omaye, 2015).

This experiment’s main goal was to compare the yield (total and marketable) of strawberry plants grown in substrate- and water-culture hydroponic systems in a greenhouse. Another objective was to quantify the inputs to the growing systems (water, energy, and footprint area) over the growth cycle and calculate resource use efficiencies for each input concerning the system output, i.e., yield. The final goal of the study was to outline the tradeoffs between systems in terms of inputs and outputs so that growers can make informed decisions in this increasingly popular CEA strawberry market. We hypothesized that varying production systems would induce distinct physiological and morphological responses in the two tested cultivars, potentially leading to improved yield and more efficient resource utilization.

## 2 Materials and methods

### 2.1 Location and environmental conditions

This experiment was conducted at the University of Georgia (College of Agricultural and Environmental Sciences, Department of Horticulture, Controlled Environment Agriculture Crop Physiology and Production lab) in Athens, Georgia, USA (latitude 33°55’55.10” N, longitude 83°21’50.51” W, altitude 198 m) from December 2022 to April 2023 in a 9.14 × 21.95 m polycarbonate greenhouse with controlled conditions.

Greenhouse air temperature and relative humidity were monitored using a digital sensor (HMP60; Vaisala, Helsinki, Finland) connected to a datalogger (CR1000X; Campbell Scientific, Logan, UT, United States) for automatic data collection. Average ± standard error day and night temperatures were 23.3 ± 0.04 and 18.2 ± 0.02°C, respectively. Day and night relative humidities were 40.5 ± 0.25 and 53.4 ± 0.24%, respectively. Vapor pressure deficits (VPD) were calculated using this temperature and relative humidity data and were 1.8 ± 0.01 and 0.96 ± 0.004 kPa for day and night, respectively. Ambient sunlight was augmented with light emitting diode (LED) fixtures (SPYDRx; Fluence Bioengineering, Austin, TX, United States), which were controlled by digital timers (Model 26898; Jasco Products LLC, Oklahoma City, OK, United States) to be activated from 4:00 PM to 7:30 PM daily. Canopy-level light was measured by a quantum sensor (SQ-610; Logan, UT, United States) connected to a separate datalogger (CR1000; Campbell Scientific, Logan, UT, United States). The sunlight and supplemental LED lighting resulted in a daily light integral (DLI) of 17.5 ± 0.67 mol·m^-2^·day^-1^.

### 2.2 Plant material

Two cultivars were used to evaluate genotypic differences, comparison of performance, broaden applicability, and identify the most suitable cultivar for each hydroponic system in CEA strawberry production.

Live plugs of ‘Florida Brilliance’ and ‘Florida Beauty’ strawberries were procured from a commercial nursery (Production Lareault Inc., Lavaltrie, QC, Canada) and arrived at the greenhouse in October 2022. Plants were watered and fertigated regularly, sorted, and transplanted into the various systems by November 2022.

### 2.3 Hydroponic systems

Strawberries were grown in four different hydroponic systems for the experiment: one substrate-culture system with plants grow in soilless media (substrate) and three water culture systems [nutrient film technique (NFT), vertical (stacked nutrient flow), and aeroponic (nutrient misted roots)].

#### 2.3.1 Substrate system

The substrate system consisted of 99.5 × 19.5 × 12.5 cm (L × W × H) troughs with a volume of 18 L (Article #7418; Beekenkamp Verpakkingen, Maasdijk, Netherlands) with eight plants each resting on 218 × 17.5 × 7 cm metal drainage gutters (B200 profile; Haygrove Limited, Ledbury, United Kingdom) with two troughs per gutter. The gutter drained into a 121 L plastic reservoir (H-3687; Uline, Pleasant Prairie, WI, United States) for recirculation. Four plants of each cultivar were placed in every trough in two contiguous lines of four. The soilless media or substrate was a 1:1 (volume:volume) mixture of super coarse perlite (Horticultural Perlite; Whittemore Co., Lawrence, MA, United States) and a peat-based commercial mix (Metro-Mix 830; Sun Gro Horticulture, Agawam, MA, United States). This product had 40-50% Canadian Sphagnum peat moss, 20-30% composted pine bark, 10-20% parboiled rice hulls, 5-10% horticultural grade vermiculite, starter nutrient charge with gypsum and slow release nitrogen (N) and wetting agent (non-organic). The substrate final substrate composition was 50% perlite, 25% peat, 15% pine bark, 7% rice hulls, and 3% vermiculite, as recommended by the Kubota Laboratory (https://u.osu.edu/indoorberry/substrates). The solution was delivered via drip irrigation with four 2.3 L·hr^-1^ emitters (Catalog no. 22000; Netafim, Tel Aviv, Israel) per trough. Regular fertigation was manually controlled with a target of 20-30% leachate per event. The four central plants in each trough were considered measurement plants, and four troughs together comprised a treatment unit.

#### 2.3.2 Nutrient film technique system

The NFT system was constructed from scratch using 243.8 × 12.7 × 12.7 cm vinyl fence posts with corresponding end caps (Barrette Outdoor Living, Middleburg Heights, OH, United States) to create channels for the nutrient solution. Eight holes were drilled in each channel for 7 cm net cups (HG3.75; Hydrofarm, Shoemakersville, PA, United States). Four plants of each cultivar were placed in each channel in an alternating pattern. A bulkhead fitting (Banjo TF-050; Alsco Industrial Products, Atlanta, GA, United States) on the down-slope side of the channel was used to enable constant recirculation, and a small length of polyvinyl chloride (PVC) pipe (4 cm) was fitted to the inner side of the bulkhead to maintain a continuous level of solution in the channel so that the bottoms of the net cups were always in contact with the solution. Four channels were built for 32 plants in the NFT system, and the central four plants in each were considered measurement plants. The nutrient solution was constantly recirculated by a submersible pump (PE-1; Little Giant, Oklahoma City, OK, United States).

#### 2.3.3 Vertical system

The vertical system was a commercially available hydroponics system (Tower Garden Flex; Tower Garden, Collierville, TN, United States). It consisted of a 75 L, bowl-shaped reservoir with a submersible pump (Syncra 3.0; Sicce, Pozzoleone, Italy) on the bottom and a columnar tower on top. This tower was made of nine 18-cm tall layers, each with four plant sites around its circumference. An inner, central column connected to the reservoir pump on the bottom reached above the top layer to the stop cap. As the pump activated, nutrient solution was sent up this column, hit the stop cap, and then percolated down through every layer into the reservoir via gravity. The area between the inner central column and the outer wall, where the plant sites were, was hollow, which allowed roots to interact with the nutrient solution as it percolated. There were also holes just below the stop cap and at every layer that aided with even distribution as the solution percolated down. The system consisted of two of these towers (one per cultivar) for a total of 72 plants, and the plants in the middle two layers of each tower were considered measurement plants. Nutrient solution delivery was controlled by a timer (TGT1; Tower Garden, Collierville, TN, United States) and ran for 5 minutes on and 45 minutes off throughout the experiment.

#### 2.3.4 Aeroponic system

The aeroponic system consisted of a mix of commercial and custom-built equipment: two black plastic tubs sloped on the inside and with grooves in the lips to hold rigid, white plastic tops with 32 pre-cut holes each for 7 × 6.25 cm (W × H) net cups (HG3.75; Hydrofarm, Shoemakersville, PA, United States). Manifolds for the inside of each tub were constructed out of PVC piping and misting nozzles (22219221202; Tefen, Kibbutz Nahsholim, Israel), which were supplied with a nutrient solution by a high-pressure pump (EF1000; Everflo Pumps, Paynesville, MN, United States). Nutrient solution delivery was controlled by the same timer as the vertical system and was on the same interval regime. The system consisted of two of these tubs (one per cultivar) for a total of 64 plants, and the plants in the middle of each tub were considered measurement plants.

### 2.4 Fertilization

A modified Yamazaki nutrient solution was used for fertigation in all systems (Kroggel and Kubota, 2017). The solution contained (all values in mg·L^-1^): 77 total N with 74 nitrate-nitrogen (NO_3_-N) and 3 ammoniacal-nitrogen (NH_4_-N), 15 phosphorous (P), 120 potassium (K), 52 calcium (Ca), 12 magnesium (Mg), 17 sulfur (S), 0.34 boron (B), 0.5 copper (Cu), 2 iron (Fe), 0.55 manganese (Mg), 0.05 molybdenum (Mo), and 0.33 zinc (Zn).

### 2.5 Fertilizer solution measurements

The reservoir solution pH and EC were measured regularly with a digital probe (Model #HI98131; Hanna Instruments, Smithfield, RI, United States) and adjusted to maintain between 5.5 and 6.5 pH and 0.75 and 1.25 dS·m^-1^, respectively. A commercial product derived from phosphoric acid was used to reduce the solution pH (pH Down; Advanced Nutrients, West Hollywood, CA, United States), while an 8M solution of potassium hydroxide was used to raise the pH. EC was lowered by diluting the solution with tap water.

### 2.6 Fruit harvest measurements

Fruit harvests were conducted every other week for December and January, then changed to every week for February through April, with 15 harvests in total. This change was instituted to accommodate the larger fruit production as the season progressed and to minimize fruit losses due to fungal pathogens. Fruit that were 70% or more ripe were harvested from each measurement plant. Fruit from each plant were counted and collectively weighed using a digital scale (Item #30430061; Ohaus Corporation, Parsippany, NJ, United States) to measure the fresh fruit yield. Marketable fruit were also counted and collectively weighed to obtain the marketable yield. A fruit was considered marketable if it weighed 8 g and was evenly pollinated. The largest marketable fruit (or simply the largest fruit if none were marketable) was cut longitudinally in half. One of the halves was weighed and then crushed using cheesecloth and a garlic press to measure total soluble solids (TSS) using a digital refractometer (Model #HI96801; Hanna Instruments, Smithfield, RI, United States). The other half, along with the remaining fruit from the plant, were placed in an 80°C oven for several days until completely dehydrated. The dehydrated fruit were weighed again to obtain the fruit dry biomass. By weighing the half-fruit used for TSS analysis, the total fruit biomass before and after dehydration was known, and thus, fruit water content could be calculated.

### 2.7 Plant harvest measurements

The strawberry plants were terminated on April 27, 2023 (129 days after transplanting). Before harvesting, the plant height was measured using a meter stick, and weighed by cutting the crown at the soil line, and weighed using a digital scale (#PB3002; Mettler Toledo, Griefensee, Switzerland) to determine fresh shoot biomass. The number of flowers, fruit, runners, and leaves was counted for each plant. Plant mortality was also assessed at this stage by direct counting. The harvest index was then calculated using the total fruit yield and the fresh shoot biomass: total fruit fresh weight ÷ (total fruit fresh weight + plant fresh weight). Next, all healthy trifoliate leaves for each plant were scanned using a leaf area meter (LI-3100; LI-COR, Lincoln, NE, USA) to obtain the total leaf area. Each plant was placed into its own paper bag and an 80°C oven for several days until completely dried. Dry shoot biomass was then measured using the same digital scale. Finally, dried trifoliate leaves were placed in sample bags and sent to a commercial lab (Waters Agricultural Laboratories, Camilla, GA, United States) for tissue nutrient concentration analysis.

Leaf N was determined by high temperature combustion process (Nelson and Sommers, 1973). Leaf P, K, Mg, Ca, S, B, Cu, Fe, Mn, and Zn concentrations were determined by inductively coupled plasma atomic emission spectrophotometer after wet acid digestion using nitric acid and hydrogen peroxide (Twyman, 2005).

### 2.8 System measurements and resource use quantification

The reservoir volumes were tracked throughout the experiment in all systems. All reservoirs were filled to a known volume at the start of the experiment and filled again to that known volume after draining and refilling. Residual reservoir volume was measured during drain and refill events, which were triggered when reservoir volume was low and/or when the reservoir pH and EC were extremely out of the ideal ranges (5.5-6.5 for pH and 0.75-1.25 for EC). By knowing reservoir volume before and after refills, total system losses from evapotranspiration (ET) were easily calculated by simple subtraction.

To calculate water use efficiency (WUE), ET per plant was first calculated by dividing reservoir ET by the number of plants supplied by that reservoir. Yield per plant was then divided by this ET per plant (based on from which system the fruit was harvested) to obtain plant WUE in grams per liter.

Total system energy use was calculated by tracking the total pump activation time in hours for each system. The NFT recirculating pump ran continuously, the leaching events in the substrate system were timed manually, and the vertical and aeroponic pumps ran on the 5-minute on and 45-minute off cycle throughout the experiment. Power consumption in Watts of all pumps was determined based on manufacturer specifications (36 W for NFT and substrate, 48 W for vertical and aeroponic). The total pump run times and power consumption rates were multiplied to obtain total system energy use in kilowatt hours (kWh).

To calculate energy use efficiency (EUE), energy use per plant was first calculated by dividing the total system energy use by the number of plants in that system. Yield per plant was then divided by this energy use per plant to obtain plant EUE in grams per kWh.

The system footprint area was calculated by measuring the widest system dimensions with a tape measure and calculating the footprint area appropriately (the substrate, NFT, and aeroponic systems’ footprint areas are rectangular, and the vertical systems are circular). One substrate trough (8 plants total), one single NFT channel (8 plants), one vertical tower (36 plants), and one aeroponic tub (32 plants) were measured for these calculations.

System area use efficiency (AUE) or the maximum yield per area that a particular system can deliver was calculated by multiplying yield per plant by the number of plants per measured system (or system component as outlined in the previous paragraph) and then dividing that resulting number by the system footprint area. Note that the AUE calculation does not consider the spacing between systems needed to implement these systems at scale effectively but instead represents an ideal maximum AUE.

### 2.9 Experimental design and statistical analysis

We tested four different hydroponic systems as the main factor: one substrate-culture system (substrate) as the control and three water culture systems NFT, vertical, and aeroponic). Each system was considered a treatment, with four replications each. The number of plants per system varied, but the same number were analyzed and measured. The substrate and the NFT system had 32 plants each, the vertical system 72, and the aeroponics system 64. Sixteen plants (two plants from two cultivars per four replications) from each system were used for measurement and analysis, while the rest were maintained and harvested from but not analyzed.

Statistical analysis was performed by conducting one-way ANOVA with Tukey’s *post-hoc* test using statistical software (SigmaPlot Version 15; Systat Software, San Jose, CA, United States) to determine significant differences among treatments. When a data set did not meet the ANOVA’s normality or equal variance conditions, a Kruskal-Wallis test with Dunn’s *post-hoc* was conducted using the same statistical software. A probability (*P*) level of 0.05 was used in all tests. Results from each cultivar were analyzed separately.

## 3 Results

### 3.1 Solution pH and EC

**Figure 1:**
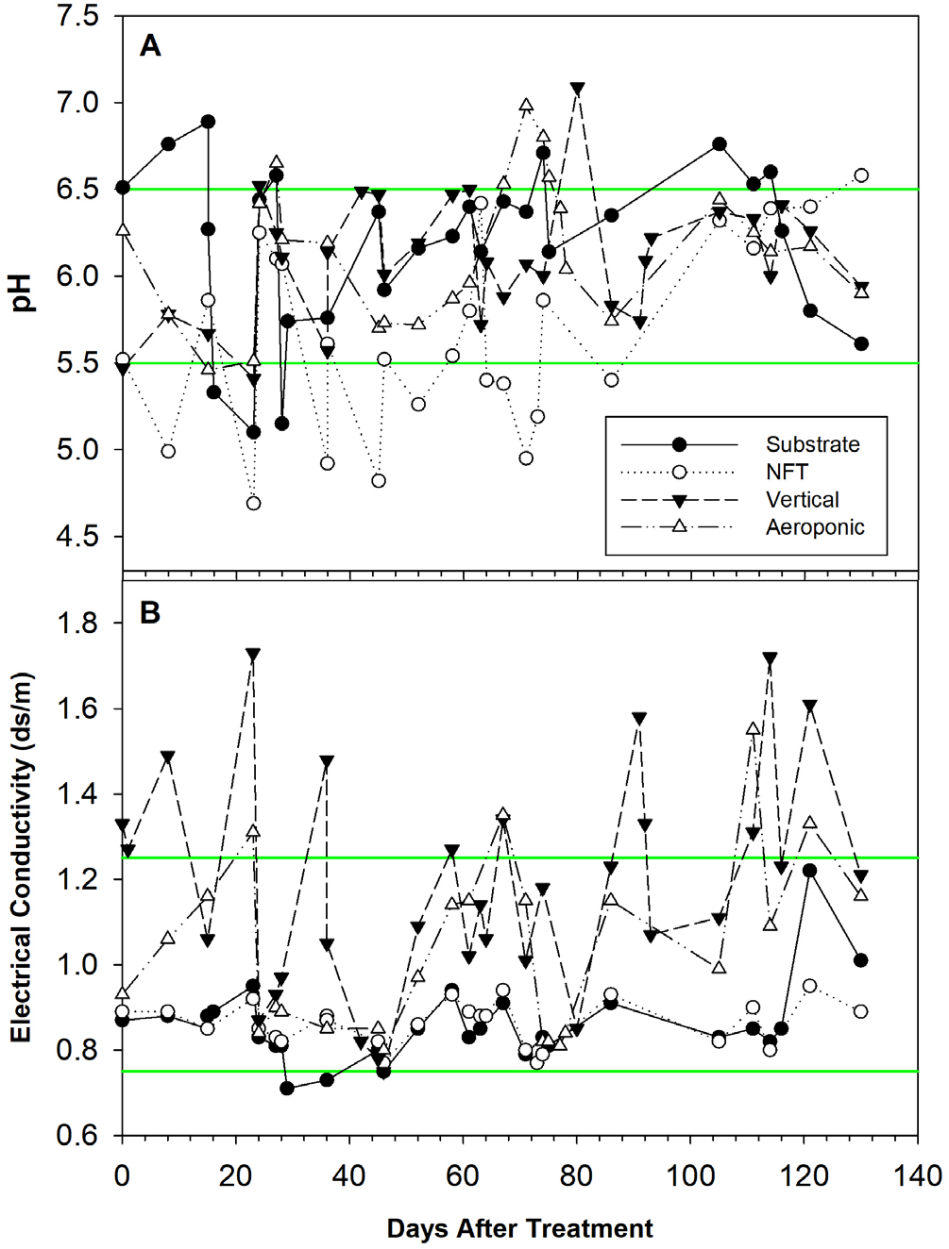
Reservoir pH **(A)** and electrical conductivity (EC) **(B)** in the substrate, nutrient film technique (NFT), vertical, and aeroponic systems from zero to 130 days after transplant. Individual data points represent pH or EC measurements. Data points for the vertical system are the average of the two towers. Green lines represent the bounds of the ideal range (5.5-6.5 for pH and 0.75-1.25 dS·m^-1^ for EC); data points between the green lines are considered within range.

The reservoir solution pH (**Figure 1A**) and EC (**Figure 1B**) varied throughout the experiment. The NFT system had the reservoir most often out of range at too acidic pH, requiring regular pH adjustment upwards. Minor deviations from the ideal pH range can be seen for all systems. The average ± standard error measured reservoir pH was 6.2 ± 0.09, 5.7 ± 0.11, 6.1 ± 0.06, and 6.1 ± 0.08 for the substrate, NFT, vertical, and aeroponic systems, respectively. The EC of the vertical system was most often out of range above the 1.25 dS·m^-1^ upper limit. Minor deviations can also be seen for the substrate and aeroponics systems, whereas the NFT system was always within the ideal EC range. The measured reservoir EC was 0.86 ± 0.017, 0.86 ± 0.010, 1.18 ± 0.047, and 1.04 ± 0.041 dS·m^-1^ for the substrate, NFT, vertical, and aeroponic systems, respectively.

### 3.2 Total and marketable fruit yield

**Figure 2:**
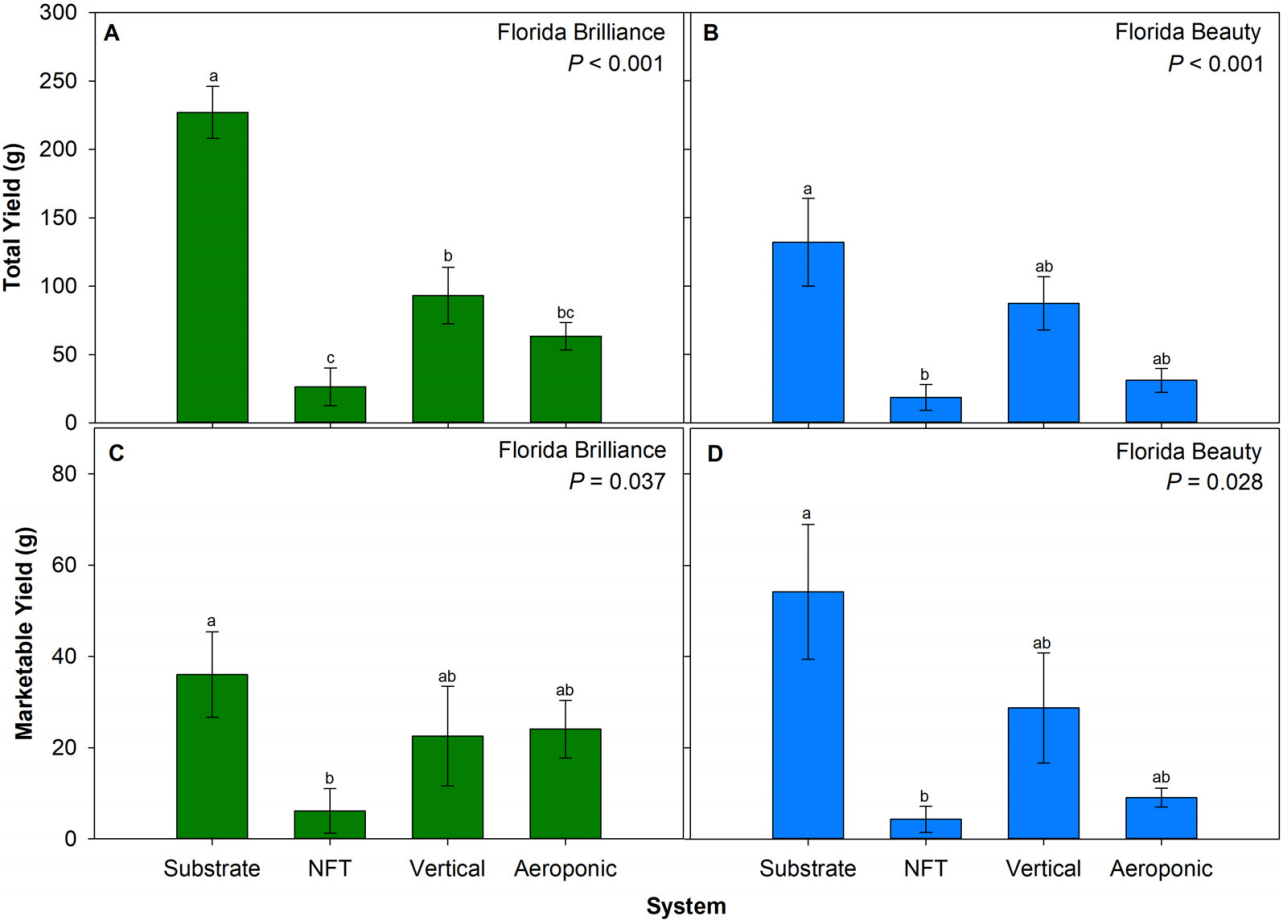
Total yield per plant for ‘Florida Brilliance’ **(A)** and ‘Florida Beauty’ **(B)**, and marketable yield per plant for ‘Florida Brilliance’ **(C)** and ‘Florida Beauty’ **(D)** in the substrate, nutrient film technique (NFT), vertical, and aeroponic systems. Each bar represents the average ± standard error of eight plants. Bars with the same letter show no significant difference; bars with different letters do show significant difference at a significance level of 5% (*P* < 0.05).

For ‘Florida Brilliance’ strawberries, the substrate system resulted in a 144% increase in yield compared to the three water culture systems, and the vertical system yield was also 71% higher than the NFT system yield (*P* < 0.001) (**Figure 2A**). The aeroponic system yield was not significantly different from the vertical or NFT systems. For ‘Florida Beauty’, the substrate system yield was 613% higher than the NFT system yield (*P* = 0.003) (**Figure 2B**). The yields from the vertical and aeroponics systems were not significantly different from either the substrate or the NFT systems. For ‘Florida Brilliance’ (*P* = 0.037) (**Figure 2C**) and ‘Florida Beauty’ (*P* = 0.028) (**Figure 2D**), the substrate system resulted in 488% and 1,160% higher marketable yield, respectively, compared to the NFT system. There were no significant differences for either cultivar when comparing the vertical and aeroponics systems to the substrate or NFT systems.

### 3.3 Fruit TSS, dry biomass, and water content

**Figure 3:**
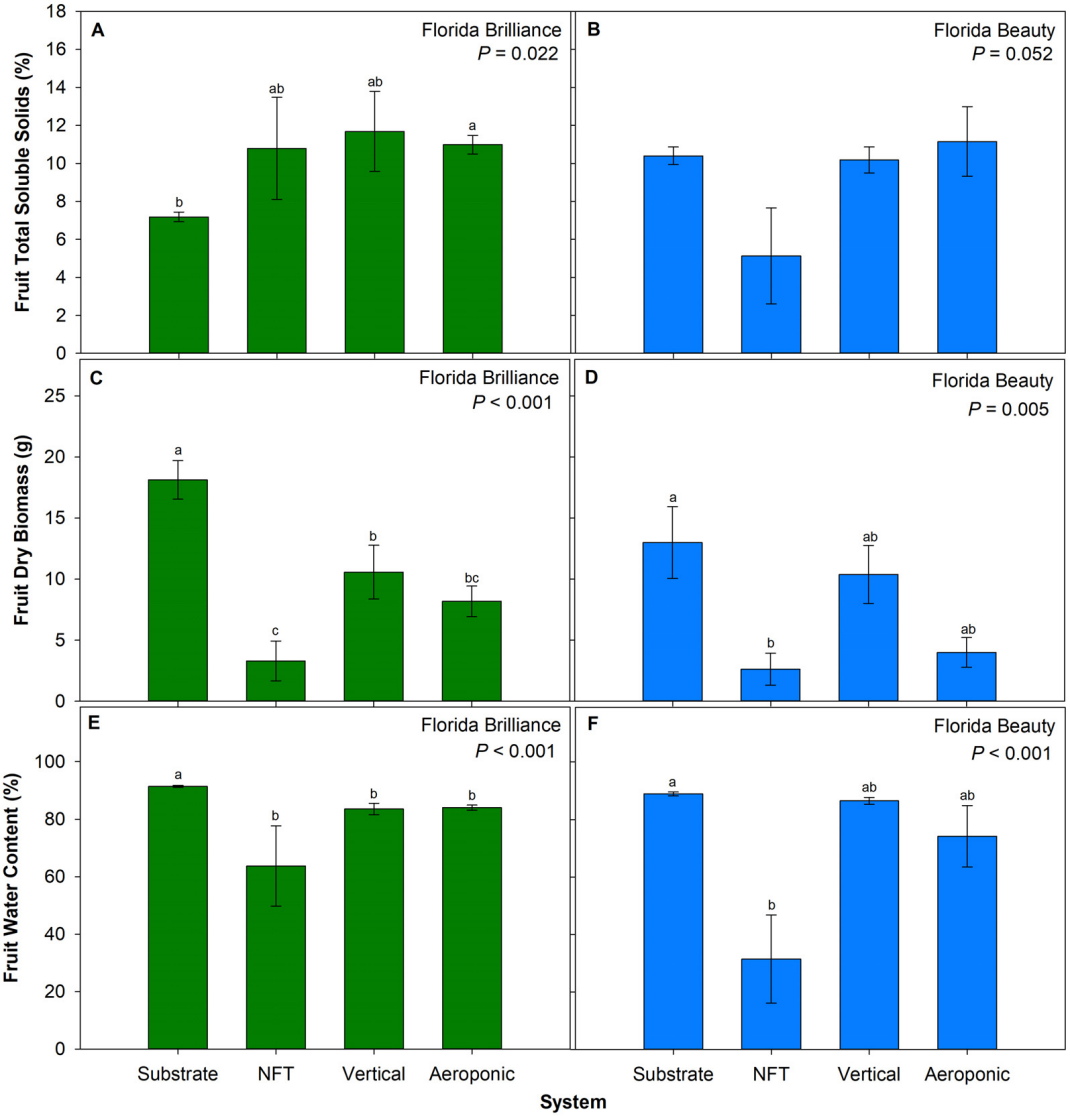
Fruit total soluble solids for ‘Florida Brilliance’ **(A)** and ‘Florida Beauty’ **(B)**, fruit dry biomass per plant for ‘Florida Brilliance’ **(C)** and ‘Florida Beauty’ **(D)**, and fruit water content for ‘Florida Brilliance’ **(E)** and ‘Florida Beauty’ **(F)** in the substrate, nutrient film technique (NFT), vertical, and aeroponic systems. Each bar represents the average ± standard error of eight plants. Bars with the same letter show no significant difference; bars with different letters do show significant difference at a significance level of 5% (*P* < 0.05).

‘Florida Brilliance’ fruit from the aeroponic system had 42% higher TSS than from the substrate system (*P* = 0.038) (**Figure 3A**). For ‘Florida Beauty’, there were no significant differences between systems for TSS (*P* = 0.052) (**Figure 3B**). The substrate system resulted in a 71% increase in fruit dry biomass for ‘Florida Brilliance’ compared to each of the three water culture systems (*P* < 0.001) (**Figure 3C**). Furthermore, the vertical system resulted in a 69% increase in fruit dry biomass compared to the NFT system. For ‘Florida Beauty’, the substrate system resulted in 395% higher fruit dry biomass than the NFT system (*P* = 0.005) (**Figure 3D**). The substrate system showed 9.4% higher fruit water content than each of the three water culture systems for ‘Florida Brilliance’ (*P* < 0.001) (**Figure 3E**). The water content for ‘Florida Beauty’ fruit grown in the substrate system was 183% higher than fruit grown in the NFT system (*P* < 0.001) (**Figure 3F**).

### 3.4 Plant height, fresh shoot biomass, and dry shoot biomass

**Figure 4:**
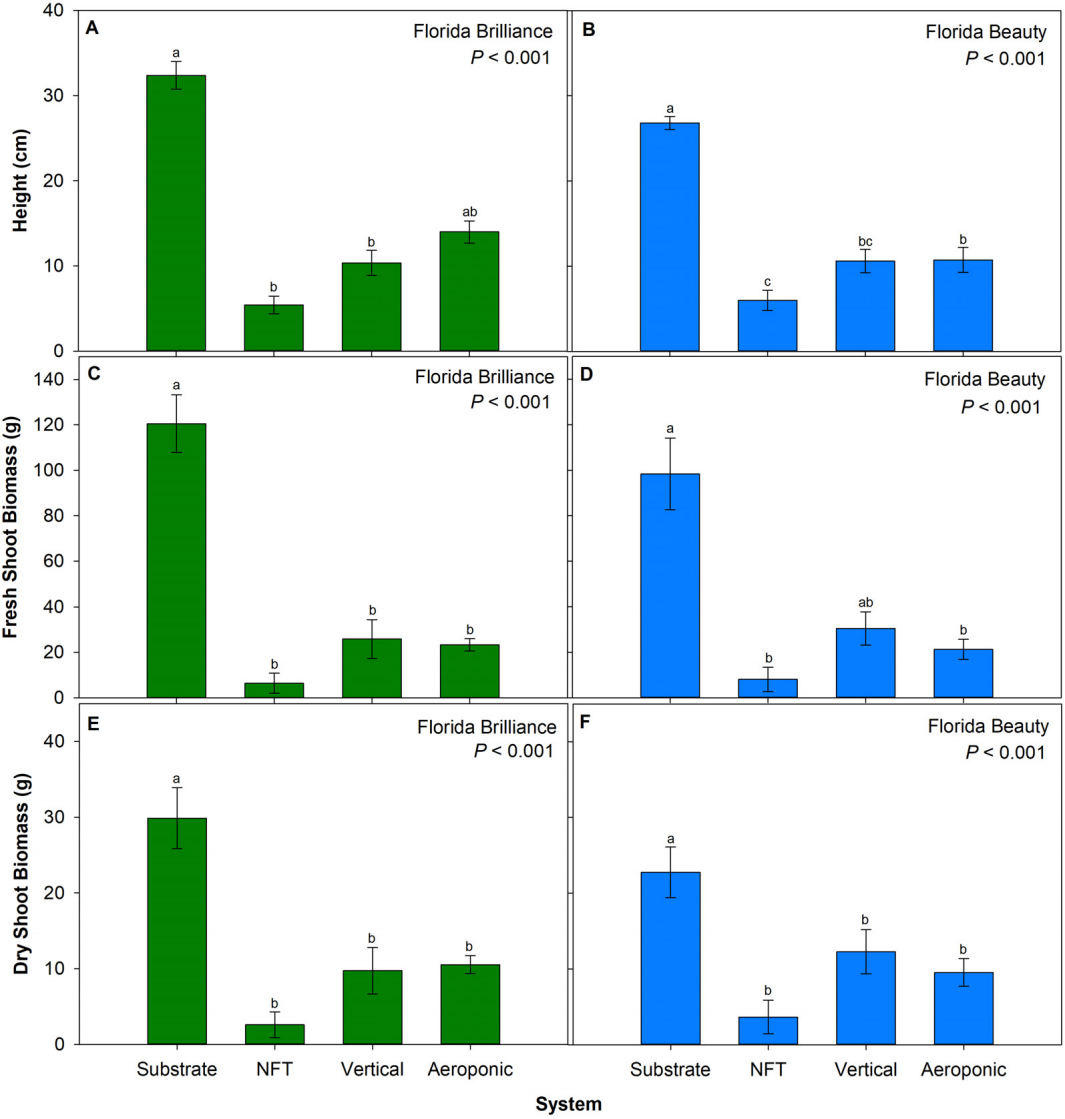
Plant height for ‘Florida Brilliance’ **(A)** and ‘Florida Beauty’ **(B)**, plant fresh shoot biomass for ‘Florida Brilliance’ **(C)** and ‘Florida Beauty’ **(D)**, and plant dry shoot biomass for ‘Florida Brilliance’ **(E)** and ‘Florida Beauty’ **(F)** in the substrate, nutrient film technique (NFT), vertical, and aeroponic systems. Each bar represents the average ± standard error of eight plants. Bars with the same letter show no significant difference; bars with different letters do show significant difference at a significance level of 5% (*P* < 0.05).

‘Florida Brilliance’ plants in the substrate system grew 203% taller compared to those in the NFT and vertical systems (*P* < 0.001) (**Figure 4A**). No significant differences were found systems between the substrate and aeroponic systems or among the three water culture systems in the’ Florida Brilliance’ plant height. The ‘Florida Beauty’ cultivar exhibited 149% taller plants in the substrate system compared to each of the three water culture systems, as well as 44% taller plants in the aeroponic system compared to the NFT system (*P* < 0.001) (**Figure 4B**). The substrate system yielded 366% higher fresh shoot biomass and 184% higher dry shoot biomass for ‘Florida Brilliance’ compared to each of the three water culture systems (both *P* < 0.001) (**Figures 4C, E**). There were no significant differences among the water culture systems for this cultivar. ‘Florida Beauty’ demonstrated 363% higher fresh shoot biomass in the substrate system compared to the NFT and aeroponic systems (*P* < 0.001) (**Figure 4D**), while there were no significant differences between the substrate and vertical systems or among the three water culture systems. The dry shoot biomass for ‘Florida Beauty’ (**Figure 4F**) was 85% higher in the substrate system compared to each of the three water culture systems, and again, there were no significant differences among the three water culture systems for that cultivar (*P* < 0.001).

### 3.5 Harvest index, leaf area, and shoot water content

**Figure 5:**
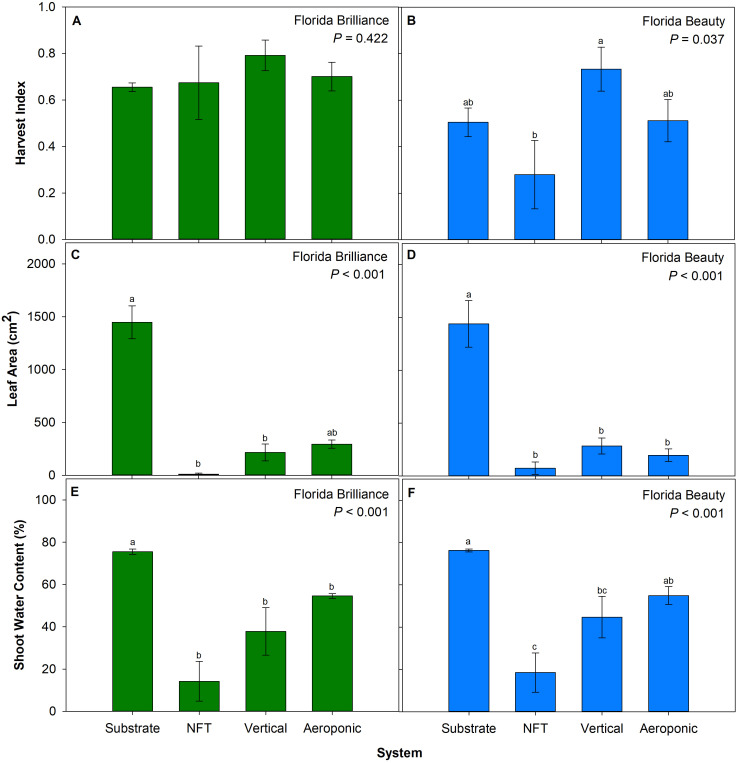
Harvest index for ‘Florida Brilliance’ **(A)** and ‘Florida Beauty’ **(B)**, leaf area per plant for ‘Florida Brilliance’ **(C)** and ‘Florida Beauty’ **(D)**, and shoot water content for ‘Florida Brilliance’ **(E)** and ‘Florida Beauty’ **(F)** in the substrate, nutrient film technique (NFT), vertical, and aeroponic systems. Each bar represents the average ± standard error of eight plants. Bars with the same letter show no significant difference; bars with different letters do show significant difference at a significance level of 5% (*P* < 0.05).

The treatments did not significantly affect the harvest index for ‘Florida Brilliance’ (*P* = 0.422) (**Figure 5A**). The harvest index for ‘Florida Beauty’ was 162% higher in the vertical system than in the NFT system (*P* = 0.037) (**Figure 5B**). There were no other significant differences among the systems. ‘Florida Brilliance’ leaf area was 571% higher in the substrate system compared to the NFT and vertical systems (*P* < 0.001) (**Figure 5C**). There were no significant differences in leaf area between the substrate and aeroponic systems or among the three water culture systems. The leaf area for ‘Florida Beauty’ was 408% higher in the substrate system compared to the three water culture systems (*P* < 0.001) (**Figure 5D**), and there were no significant differences in leaf area among the three water culture systems. ‘Florida Brilliance’ shoot water content showed a 38% increase in the substrate system compared to the three water culture systems (P < 0.001) (**Figure 5E**). ‘Florida Beauty’ shoot water content in the substrate system showed a 71% increase compared to the vertical and NFT systems. Furthermore, the aeroponic system showed a 197% increase in shoot water content compared to the NFT system (P < 0.001) (**Figure 5F**).

### 3.6 Leaf tissue macronutrient concentration

‘Florida Brilliance’ leaf K was 32% higher in the vertical system compared to the substrate system (*P* = 0.043) (**Table 1**). Similarly, leaf S in the vertical system was 38% higher for this cultivar than in the substrate system (*P* = 0.002). ‘Florida Brilliance’ leaf N (*P* = 0.903), P (*P* = 0.220), Mg (*P* = 0.642), and Ca (*P* = 0.059) were not significantly affected by the treatments. ‘Florida Beauty’ leaf Mg was 71% higher in the substrate system compared to the vertical system (*P* = 0.004) (**Table 1**). Leaf N (*P* = 0.056), P (*P* = 0.114), K (*P* = 0.667), Ca (*P* = 0.615), and S (*P* = 0.667) for ‘Florida Beauty’ were not significantly affected by the treatments.

**Table 1:** ‘Florida Brilliance’ and ‘Florida Beauty’ strawberry (*Fragaria × ananassa*) average leaf macronutrient concentrations in the substrate, nutrient film technique (NFT), vertical, and aeroponic systems.

Cultivar	System	Nitrogen (%)	Phosphorus (%)	Potassium (%)	Magnesium (%)	Calcium (%)	Sulphur (%)
Florida Brilliance	Substrate	2.38 ± 0.066	0.43 ± 0.025	2.31 ± 0.060 b	0.35 ± 0.020	1.44 ± 0.057	0.16 ± 0.005 b
	NFT	2.37*	0.71*	3.15*	0.25*	1.16*	0.23*
	Vertical	2.42 ± 0.081	0.53 ± 0.079	3.04 ± 0.248 a	0.32 ± 0.042	1.95 ± 0.164	0.22 ± 0.003 a
	Aeroponic	2.36 ± 0.108	0.58 ± 0.056	2.66 ± 0.155 ab	0.32 ± 0.012	1.62 ± 0.142	0.19 ± 0.011 ab
*P*		0.903	0.220	0.043	0.642	0.059	0.002
Florida Beauty	Substrate	2.55 ± 0.084	0.66 ± 0.019	2.59 ± 0.088	0.41 ± 0.006 a	1.60 ± 0.126	0.18 ± 0.005
	NFT	2.41 ± 0.080*	0.53 ± 0.050*	3.30 ± 0.875*	0.33 ± 0.025*	1.43 ± 0.365*	0.30 ± 0.110*
	Vertical	2.26 ± 0.066	0.46 ± 0.104	2.75 ± 0.091	0.24 ± 0.036 b	1.77 ± 0.194	0.20 ± 0.024
	Aeroponic	2.25 ± 0.101	0.54 ± 0.044	2.75 ± 0.184	0.34 ± 0.025 ab	1.59 ± 0.068	0.19 ± 0.009
*P*		0.056	0.114	0.667	0.004	0.615	0.667
Test		A	KW	KW	A	A	KW

For ‘Florida Brilliance’, statistical analysis was conducted using one-way ANOVA and Tukey’ HSD. For ‘Florida Beauty’, statistical analysis for leaf N, Mg, and Ca was conducted using one-way ANOVA and Tukey’s HSD (A), while the Kruskal-Wallis test with Dunn’s *post-hoc* (KW) was used for leaf P, K, and S due to invalid ANOVA assumptions. All tests executed using significance level of 5% (*P* < 0.05). Values for the NFT system were excluded from analysis due to high mortality and low sample count (*).

### 3.7 Leaf tissue micronutrient concentration

Both leaf Zn (*P* = 0.002) and Cu (*P* < 0.001) for ‘Florida Brilliance’ were 119% and 235% higher, respectively, in the vertical system compared to the substrate system (**Table 2**). Conversely, leaf Fe in the substrate system was 413% higher than in the vertical system for this cultivar (*P* = 0.001). Finally, the ‘Florida Brilliance’ leaf Mn was 112% higher in the vertical system compared to the aeroponics system (*P* = 0.006). ‘Florida Brilliance’ Leaf B was also significantly affected by the treatments, however the *post-hoc* test could not distinguish between treatments (*P* = 0.030). ‘Florida Beauty’ leaf B was 40% higher in the vertical system compared to the substrate and aeroponic systems (*P* = 0.003) (**Table 2**). Both leaf Fe (*P* = 0.015) and leaf Cu (*P* = 0.015) for this cultivar were significantly affected by the treatments, however the *post-hoc* tests could not distinguish between treatments. The treatments did not significantly affect ‘Florida Beauty’ leaf Zn (P = 0.181) and Mn (P = 0.364).

**Table 2:** ‘Florida Brilliance’ and ‘Florida Beauty’ strawberry (*Fragaria × ananassa*) average leaf micronutrient concentrations in the substrate, nutrient film technique (NFT), vertical, and aeroponic systems.

Cultivar	System	Boron (mg/kg)	Zinc (mg/kg)	Manganese (mg/kg)	Iron (mg/kg)	Copper (mg/kg)
Florida Brilliance	Substrate	151.50 ± 3.663 a	18.00 ± 0.816 b	187.75 ± 22.287 ab	514.00 ± 58.242 a	4.25 ± 0.250 b
	NFT	141.00*	36.00*	118.00*	91.00*	7.00*
	Vertical	246.75 ± 26.750 a	39.50 ± 6.461 a	235.50 ± 24.767 a	123.50 ± 9.682 ab	14.25 ± 1.493 a
	Aeroponic	157.00 ± 20.547 a	30.00 ± 2.082 ab	111.00 ± 12.537 b	100.25 ± 8.159 b	10.00 ± 0.707 ab
*P*		0.030	0.002	0.006	0.001	<0.001
Test		KW	KW	A	KW	KW
Florida Beauty	Substrate	129.00 ± 11.881 b	23.00 ± 1.683	203.25 ± 9.801	517.00 ± 86.374 a	4.00 ± 0.408 a
	NFT	126.00 ± 1.000*	29.00 ± 2.000*	223.50 ± 109.500*	141.50 ± 29.500*	10.50 ± 1.500*
	Vertical	225.00 ± 21.486 a	30.25 ± 5.452	205.75 ± 38.042	115.00 ± 26.805 a	15.00 ± 5.730 a
	Aeroponic	161.25 ± 2.496 b	38.00 ± 6.964	157.25 ± 20.782	97.25 ± 9.232 a	10.00 ± 1.000 a
*P*		0.003	0.181	0.364	0.015	0.015
Test		A	A	A	KW	KW

For ‘Florida Brilliance’, statistical analysis for leaf Mn was conducted using one-way ANOVA and Tukey’s HSD (A), while the Kruskal-Wallis test with Dunn’s *post-hoc* (KW) was used for leaf B, Zn, Fe, and Cu due to invalid ANOVA assumptions. For ‘Florida Beauty’, statistical analysis for leaf B, Zn, and Mn was conducted using one-way ANOVA and Tukey’s HSD (A), while the Kruskal-Wallis test with Dunn’s *post-hoc* (KW) was used for leaf Fe and Cu due to invalid ANOVA assumptions. All tests executed using significance level of 5% (*P* < 0.05). Values for the NFT system were excluded from analysis due to high mortality and low sample count (*).

### 3.8 Resource use efficiency

**Figure 6:**
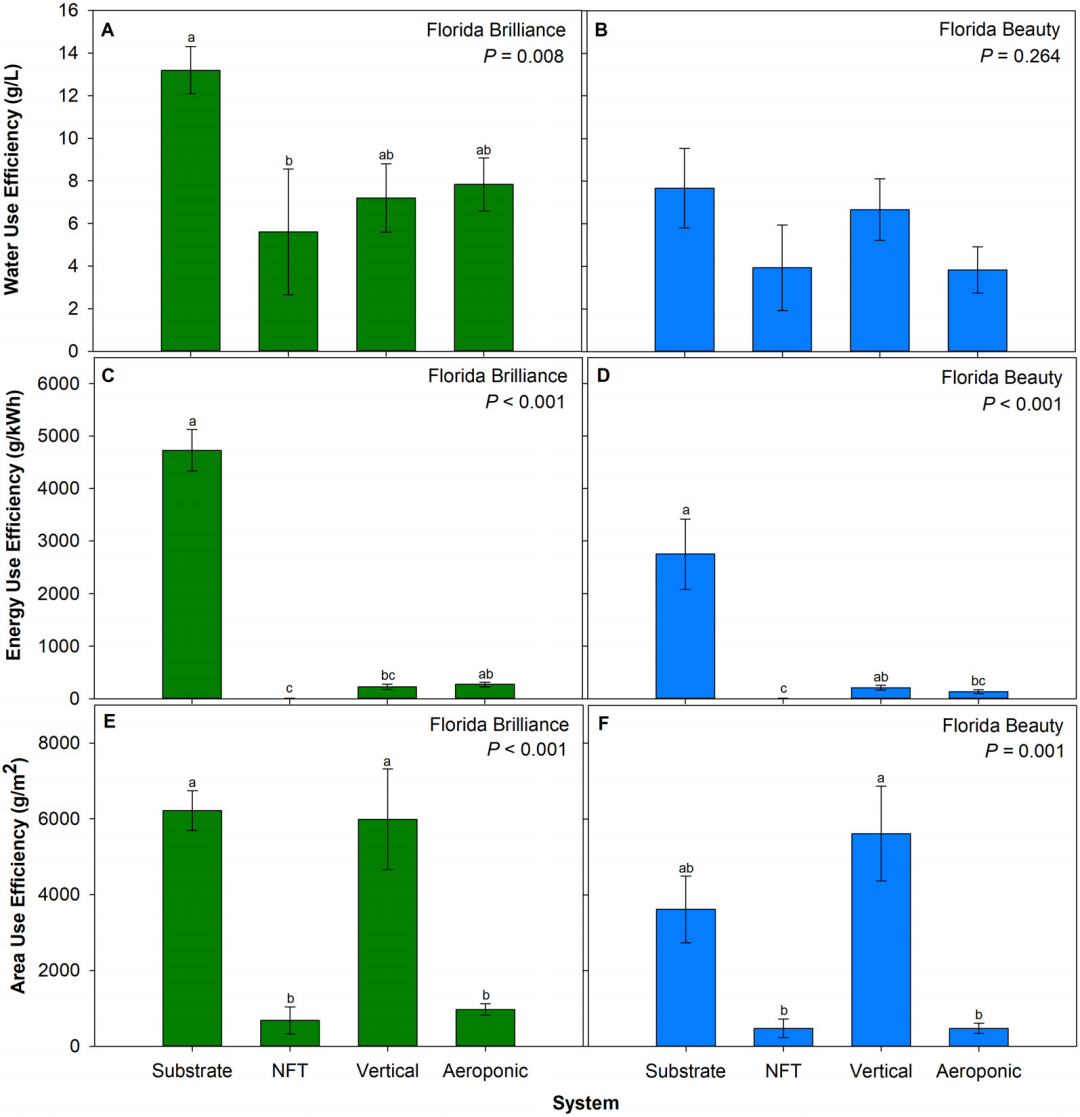
Water use efficiency for ‘Florida Brilliance’ **(A)** and ‘Florida Beauty’ **(B)**, energy use efficiency for ‘Florida Brilliance’ **(C)** and ‘Florida Beauty’ **(D)**, and area use efficiency or maximum yield per area that a particular system can deliver for ‘Florida Brilliance’ **(E)** and ‘Florida Beauty’ **(F)** in the substrate, nutrient film technique (NFT), vertical, and aeroponic systems. Each bar represents the average ± standard error of eight plants. Bars with the same letter show no significant difference; bars with different letters do show significant difference at a significance level of 5% (*P* < 0.05).

‘Florida Brilliance’ WUE was 135% higher in the substrate system than in the NFT system (*P* = 0.008) (**Figure 6A**). The treatments did not significantly affect ‘Florida Beauty’ WUE (*P* = 0.264) (**Figure 6B**). EUE for ‘Florida Brilliance’ was 1,996% higher in the substrate system than in both the vertical and NFT systems (*P* < 0.001) (**Figure 6C**). EUE in the aeroponic system for this cultivar was also 3,514% higher than in the NFT system. Similarly, ‘Florida Beauty’ EUE in the substrate system was 1,957% higher than in both the NFT and aeroponic systems (*P* < 0.001) (**Figure 6D**). The EUE in the vertical system was also 3,880% higher than the EUE in the NFT system for this cultivar. ‘Florida Brilliance’ AUE was 320% higher in both the substrate and vertical systems than in the NFT and aeroponic systems (*P* < 0.001) (**Figure 6E**). For ‘Florida Beauty’, the vertical system had 1,074% higher AUE than the NFT and aeroponic systems (*P* = 0.001) (**Figure 6F**).

## 4 Discussion

There were similar trends in both strawberry cultivars for total fruit yield per plant. The substrate system had the highest yield, followed by the vertical, aeroponic, and NFT systems in that order (**Figures 2A, B**).

The precise cause of the substrate system’s higher fruit yield is unclear; however, our results match those of a previous study that observed an increase in strawberry yield from a soil system compared to NFT and aeroponic system yields (Albaho et al., 2008). Another study reported higher yield from strawberry plants grown in a water culture system than in a soil system; however, that study had high plant mortality and significant pest and disease issues that negatively impacted soil system yields (Treftz and Omaye, 2015). It is not likely that this difference arose due to the substrate system providing greater access to root zone oxygen; both the vertical and aeroponic systems allowed plant roots to be exposed to the ambient atmosphere for 45 out of every 50 minutes during the experiment. Since the harvest index results were not extraordinarily affected by the systems (**Figures 5A, B**), we can infer that a general increase in biomass production resulted in higher substrate system yields. We observed that strawberry plants grown in the substrate system were the tallest (**Figures 4A, B**), had the greatest fresh and dry shoot biomass (**Figures 4C-F**), and had the greatest leaf area (**Figures 5C, D**). Larger plants with greater leaf areas can absorb more light and produce more photosynthates. Since fruit is a carbohydrate sink organ in the plant, greater availability of carbohydrates from photosynthesis should lead to more and larger fruit development. This positive correlation between the vegetative biomass and yield has also been reported previously in aeroponic, substrate, and soil strawberry production systems (Akon et al., 2018; Madhavi et al., 2023, 2021). The most likely explanation for this increase in overall biomass in the substrate system is that those plants were less stressed than plants in the water culture systems. We observed that both cultivars had the highest shoot water content in the substrate system (**Figures 5E, F**). Leaf growth is driven by cell water uptake and storage to increase turgor pressure and expand cell walls (Boyer, 1988). As leaves become more fully developed and expanded, mesophyll cells contain more water, and thus shoot water content increases. Reductions in shoot water content due to stress factors such as heat and drought have been previously correlated with reductions in biomass and yield for barley (*Hordeum vulgare*), bell pepper (*Capsicum annum*), maize (*Zea mays sinensis* ‘Kulesh’), and snap bean (*Phaseolus vulgaris*) (Behbahanizadeh et al., 2014; Ferrara et al., 2011; Kumar et al., 2006; Yang et al., 2020). We observed the same correlation in both strawberry cultivars, indicating that the plants in the water culture systems were likely experiencing an increase in osmotic stress that led to reduced biomass accumulation.

A possible contributing factor to the increased biomass production in the substrate system could be the greater mechanical stability provided by the system. Although root biomass was not measured in this study, roots grown into substrate should theoretically afford greater mechanical stability than roots grown into a liquid solution. This increased mechanical stability could allow plants to grow taller and produce more vegetative biomass. Another possible contributing factor for the greater growth and yield in the substrate system is the increase in iron uptake in the substrate system because of the substrate cation exchange capacity. Iron is vital to the electron transport process during photosynthesis due to its inclusion in photosystem II, cytochrome *b6f*, photosystem I, and ferredoxin. Without enough iron available within leaf mesophyll cells, the entire photosynthetic machinery and downstream biological processes would be slowed (Briat et al., 2015; Taiz and Zeiger, 2010). We observed much greater iron foliar concentrations for both cultivars in the substrate system compared to the other three systems, with the substrate system measuring over 500 mg·kg^-1^ of iron and the water culture systems all measuring between 90 and 150 mg·kg^-1^ (**Tables 1**, **2**). However, the range of 90 to 150 mg·kg^-1^ foliar iron is not necessarily deficient. No symptoms of iron deficiency, such as interveinal chlorosis, were observed during the study, indicating that chlorophyll biosynthesis was not inhibited at those concentrations. This observation agrees with previous research that has listed sufficiency ranges for strawberry foliar iron as 50-3000, 50-100, 50-300, and 102-188 mg·kg^-1^ (Bottoms et al., 2013). Our observed values of foliar iron in the substrate system exceeded three out of four of these ranges. It could be possible that the foliar iron concentrations in the water culture systems were sufficient to prevent chlorosis from developing but resulted in a slow electron transfer process. Biochemical analyses would be needed to investigate this response further.

The treatments also affected fruit quality, though less than the total yield. Marketable yield followed a similar pattern in both cultivars to total yield: the substrate system had the greatest marketable yield, with the vertical and aeroponic systems next and the NFT system last. However, marketable yield was low in all systems, with no system yielding more than an average of 60 g per plant. This was primarily due to a potential lack of thorough pollination inside the greenhouse. Thorough pollination of strawberry flowers is essential for fruit to develop a symmetric, marketable shape. Strawberry flowers can self-pollinate, but biotic pollinators have been shown to improve pollination and produce larger, more evenly shaped fruit (Gudowska et al., 2024). We could not use biotic pollinators for this study and relied on active (deliberate with hands and blowers) and passive (from ambient air circulation) mechanical pollination. This likely led to reduced marketable yields.

The measurement of TSS by refractometry is a well-established approximation of the sugar concentration of strawberry fruit (Madhavi et al., 2023). Only the cultivar Florida Brilliance produced fruit with significantly different TSS in the hydroponic systems, with the substrate system producing the lowest average TSS (**Figures 3A, B**). This low TSS from the substrate system for ‘Florida Brilliance’ matches the TSS values reported by a previous study for field trials of the same cultivar from February 2016 through March 2018, which ranged from 5.73-8.46% (Whitaker et al., 2019). TSS values from the three water culture systems from this cultivar all exceeded this range. These results match those from a previous study that reported increases in strawberry fruit TSS when plants were subjected to drought stress (Yenni et al., 2022). ‘Florida Brilliance’ fruit from the substrate system also had significantly higher fruit water content than the three water culture systems; this increase in fruit water content alone could account for the decrease in TSS in fruit from the substrate system.

WUE was significantly affected by the treatments only for the cultivar Florida Brilliance, with the substrate system having the highest WUE (**Figures 6A, B**). This is an unexpected result because it has been shown that for other commonly grown hydroponic crops, such as lettuce (*Lactuca sativa*), water culture systems have previously resulted in greater WUE than substrate systems (Majid et al., 2021). This higher WUE in the substrate system is driven primarily by its larger yield. The average ET per plant in the substrate system was 17.21 L; in the NFT system, it was 4.70 L; in the aeroponic system, it was 8.10 L; and in the vertical system, it was 12.48 L. The substrate system resulted in the largest ET per plant due to higher evaporation and transpiration than the water culture systems. There was higher evaporation in the substrate system because as a substrate is irrigated, some of the water will evaporate before the plant can take it up. The higher transpiration resulted from those plants having higher leaf areas and, thus, more stomata for transpiration. Even though the substrate system had the highest ET per plant, the yield from the substrate system was large enough to compensate for this higher ET and result in the largest WUE.

The substrate system also had the highest EUE, which is expected (**Figures 6C, D**). In water culture systems, a pump must run for plants to access water and nutrients. In a substrate system, the substrate retains water and nutrients provided during fertigation events that plants can then uptake over time. The average energy consumption per plant was 3.483 kWh in the NFT system, 0.413 kWh in the vertical system, 0.232 kWh in the aeroponic system, and 0.048 kWh in the substrate system. The energy consumption was so high in the NFT system due to the pump running continuously. Both the vertical and aeroponic systems ran for 5 minutes out of every 50 minutes, which is 10% of the time, and hence why the energy consumption from these two systems is approximately one order of magnitude less than the NFT system energy consumption. The substrate system had the lowest energy consumption with a total pump run time of 42.65 hours out of the 3096-hour (129-day) long experiment. These order-of-magnitude differences in energy consumption per plant are the primary drivers behind the differences in EUE among the systems.

The AUE analysis produced perhaps the most interesting results and demonstrated that water culture systems, specifically the vertical system, may have some relevant applications. The vertical system had an AUE extremely close to the substrate system AUE for the cultivar Florida Brilliance, whereas for ‘Florida Beauty’, the vertical system AUE was larger than the substrate system AUE (**Figures 6E, F**). These AUE values are primarily driven by the vertical system’s ability to maximize planting density. The vertical system had 64.3 plants per meter, the substrate system 27.4 plants per meter, the NFT system 25.8 plants per meter, and the aeroponic system 15.3 plants per meter. The vertical and substrate systems for both cultivars had AUE values between 3 and 6.5 kilograms per square meter (kg·m^-2^), which match with the results from a previous study that reported yields of 3.1, 5.5, and 6.5 kg·m^-2^ for the strawberry cultivar Albion grown in a greenhouse substrate hydroponic system during three different seasons in both Arizona and Ohio (McKean, 2019).

## 5 Conclusions

The substrate system produced the highest yields on a per-plant basis. The substrate system also utilized water and energy most efficiently to produce these high yields, and the substrate and vertical systems had high-efficiency use of the growing area. Our results show that the substrate system is the optimal growing system for hydroponic strawberries in greenhouses. It is possible through further research and refinement that water culture systems could be commercially viable, particularly systems that maximize planting density, such as the vertical system in this study. However, the area utilized for this trial was limited, and the results may not be directly applicable to a commercial scale operation. Further testing is necessary to evaluate scalability.

## Data Availability

The raw data supporting the conclusions of this article will be made available by the authors, upon reasonable requests.
